# Supplementary figures and images for: The diverse radiodont fauna from the Marjum Formation of Utah, USA (Cambrian: Drumian)
Source: PeerJ. 2021 Jan 19;9:e10509. doi: 10.7717/peerj.10509 (PMC7821760; doi:10.7717/peerj.10509)

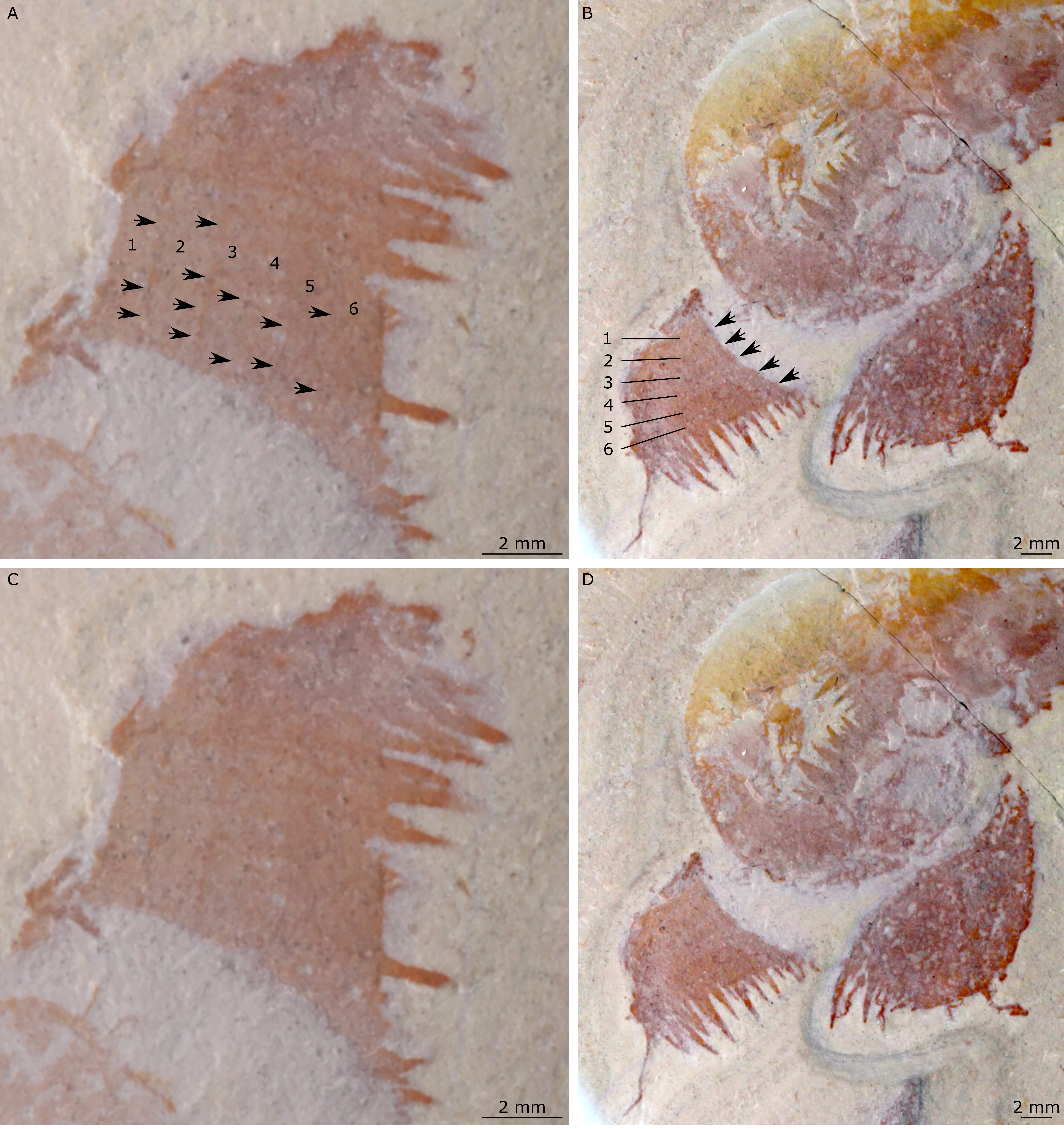

Supplement: Supplemental Information 1 — (A, C) BPM 1108b, counterpart of holotype. (B, D) BPM 1108a, part of holotype. Numbers indicate plate-like endites, numbered from proximal to distal. Arrows indicate boundaries between endites. [file peerj-09-10509-s001.png]
